# In Silico Identification of New Anti-SARS-CoV-2 Main Protease (M^pro^) Molecules with Pharmacokinetic Properties from Natural Sources Using Molecular Dynamics (MD) Simulations and Hierarchical Virtual Screening

**DOI:** 10.1155/2022/3697498

**Published:** 2022-10-10

**Authors:** Harrison Onyango, Patrick Odhiambo, David Angwenyi, Patrick Okoth

**Affiliations:** ^1^Department of Biological Sciences (Molecular Biology, Computational Biology and Bioinformatics Section), School of Natural and Applied Sciences, Masinde Muliro University of Science and Technology, P. O BOX 190, Kakamega 50100, Kenya; ^2^Department of Mathematics, School of Natural and Applied Sciences, Masinde Muliro University of Science and Technology, P. O BOX 190, Kakamega 50100, Kenya

## Abstract

Infectious agents such as SARS-CoV, MERS-CoV, and SARS-CoV-2 have emerged in recent years causing epidemics with high mortality rates. The quick development of novel therapeutic compounds is required in the fight against such pathogenic agents. Unfortunately, the traditional drug development methods are time-consuming and expensive. In this study, computational algorithms were utilized for virtual screening of a library of natural compounds in the ZINC database for their affinity towards SARS-CoV-2 M^pro^. Compounds such as cinanserin, nelfinavir, baicalin, baicalein, candesartan cilexetil, chloroquine, dipyridamole, and hydroxychloroquine have the ability to prevent SARS-CoV-2 M^pro^ from facilitating COVID 19 infection; thus, they treat COVID 19. However, these drugs majorly act to reduce the symptoms of the disease. No anti-viral drug against COVID 19 virus infection has been discovered and approved. Therefore, this study sought to explore natural inhibitors of SARS-CoV-2 M^pro^ to develop a pharmacophore model for virtual screening of natural compounds in the ZINC database as potential candidates for SARS-CoV-2 M^pro^ inhibitors and as therapeutic molecules against COVID 19. This study undertook in silico methods to identify the best anti-viral candidates targeting SAR-CoV-2 M^pro^ from natural sources in the ZINC database. Initially, reported anti-SARS-CoV-2 M^pro^ molecules were integrated into designing a pharmacophore model utilizing PharmaGist. Later, the pharmacophore model was loaded into ZINCPHARMER and screened against the ZINC database to identify new probable drug candidates. The root means square deviation (RMSD) values of the potential drug candidates informed the selection of some of them, which were docked with SARS-CoV-2 M^pro^ to comprehend their interactions. From the molecular docking results, the top four candidates (**ZINC000254823011**, **ZINC000072307130**, **ZINC000013627512**, and **ZINC000009418994**) against SARS-CoV-2 M^pro^, with binding energies ranging from –8.2 kcal/mol to –8.6 kcal/mol, were examined for their oral bioavailability and other pharmacokinetic properties. Consequently, **ZINC000072307130** emerged as the only orally bioavailable drug candidate with desirable pharmacokinetic properties. This candidate drug was used to perform MD simulations, and the outcomes revealed that **ZINC000072307130** formed a stable complex with the viral main protease. Consequently, **ZINC000072307130** emerges as a potential anti-SARS-CoV-2 M^pro^ inhibitor for the production of new COVID 19 drugs.

## 1. Introduction

SARS-CoV-2 infection originated in Wuhan, the first case reported in December 2019. By December 2020, more than 1.4 million individuals had died from the sickness, and over 6.35 million persons had contracted the disease [1]. SARS-CoV-2 has continually menaced human health, causing significant morbidity and mortality globally. Patel et al. [2] explain that the virus can spread by various routes, including animal-to-human transmission, mother-to-child, sexual intercourse, ocular, bloodborne, fecal-oral, direct contact, and airborne. Even though SARS-CoV-2 primarily causes a moderate respiratory infection, many individuals develop severe sickness that leads to death [3]. In addition, many asymptomatic diseases can spread the virus to others. COVID 19 patients who have underlying illnesses are predisposed to contracting a severe illness [4].

The Middle East respiratory syndrome-related coronavirus (MERS-CoV), severe acute respiratory syndrome coronavirus (SARS-CoV), and SARS-CoV-2 are among the strains of COVID that cause infections in people and animals [5]. SARS-CoV-2, the etiological COVID 19 agent, has a 78% genetic similarity to SARS-CoV, the virus that caused the 2003 SARS outbreak [6]. It causes infection by interacting with receptors and transmembrane proteases on the cell membrane of the host. El-Ashrey et al. [7] and Hoffmann et al. [8] point out that the virus interacts with angiotensin-converting enzyme 2 (ACE2) transmembrane protease and receptor serine 2 (TMPRSS2) to cause infection.

The virus enters host cells by attaching to ACE2 on cell membranes, resulting in immune reactions and inflammation [1]. After binding, the interaction between the spike glycoprotein and cellular proteases leads to cleaving and the virus's subsequent entry into the cell [9, 10]. The viral genome is then released into the cytosol. The host cell machinery translates it, producing RNA-dependent RNA polymerase (RdRp), helicase enzymes, and viral proteases [1]. RdRp has a vital role in replicating viral genomes and the translation of structural proteins [10]. Identifying drug targets within the scope of the virus mechanism of action is essential in identifying effective anti-virals.

Because it differs from human proteases, the viral M^pro^ enzyme is believed to be a prospective therapeutic target. Currently, drugs such as remdesivir, molnupiravir, fluvoxamine, and paxlovid have been reported as anti-viral treatments for COVID 19 [11]. However, only remdesivir is FDA-approved to treat COVID 19 [12]. Regardless, it is not widely used because some clinical trials never proved its advantageous impacts on SARS-CoV-2. Similarly, remdesivir is expensive and requires intravenous administration in hospitals [11]. Therefore, it is crucial to develop simple oral COVID 19 drugs. The limited number of approved anti-COVID 19 drugs continues to be a challenge to several scientists globally. SARS-Cov-2 M^pro^ is among the integral targets for COVID 19 drug production since its maturation and that of other important polyproteins after the viral spike protein binds to angiotensin-converting enzyme 2 (ACE2) receptor lead to the virus's entry into the host cell and subsequent COVID 19 infection. It is essential for the virus's proteolytic development [7]. It is thought to be a probable target for diminishing the spread of the illness by blocking the active areas of viral polyprotein cleavage [7]. M^pro^'s sequence and structure are very similar to those of other beta coronaviruses. C-terminal domain-III, N-terminal domain-II, and N-terminal domain-I make up the M^pro^ monomer. M^pro^'s active region has a catalytic dyad containing His41 and Cys145 [13].

The virus's complex and shifting nature piqued the interest of several researchers from various fields around the globe. The researchers integrated their endeavors to combat the pandemic by reexamining the conceivable side effects of presently available treatments, evaluating passive immunity, and searching for vaccines. Currently, several vaccines have been manufactured to reduce the harmful effects of the virus on human health. Similarly, the potency of several drugs to treat COVID 19 is presently being explored. Tumban [10] outlines that Veklury (remdesivir) was recently licensed by the US Food and Drug Administration (FDA) to treat 12-year-old COVID 19 patients weighing a minimum of 40 kgs. Remdesivir binds to RdRp, barring the viral DNA from replicating [10]. To control respiratory dysfunction, the key remedy is symptomatic and oxygen therapy. Mechanical ventilation is suggested in the event of respiratory failure to avoid respiratory arrest. Intensive care is usually required in the case of complex diseases due to multiple organ failure (MOF) or acute respiratory distress syndrome (ARDS) [3].

Anti-virals (remdesivir, lopinavir enhanced with ritonavir, chloroquine and hydroxychloroquine, and bemcentinib) are being studied extensively to see if they may be used to treat COVID 19. COVID 19 treatment is medically unmet; thus, developing viable medications to stop infection and disease development is crucial [3]. Drugs that operate directly on conserved enzymes such as M^pro^ could have a broad spectrum of action and be effective [14]. The main challenge in developing a medicine is that attacking SARS-CoV-2 without causing aftereffects in the host is extremely difficult. The virus relies on its host to survive and multiply; hence, most biochemical pathways are identical [15]. Similarly, the de novo drug design method can lead to less-effective SARS-CoV-2 M^pro^ inhibitors and be time-consuming [16]. Therefore, bioinformatics and computational biology tools have been primarily employed to identify or discover molecules of known structures that are SARS-CoV-2 M^pro^ inhibitors. The association between the protein (SARS-Cov-2 Mpro) and the ligand helps comprehend the actual pharmacological mechanism. Nature is still a suitable option for renewable sources of drugs utilized to deal with numerous emerging health issues.

Computational biology and bioinformatics provide a time- and cost-effective option for developing promising lead molecules. Computational techniques such as virtual screening and molecular dynamics (MD) have been investigated to explore and recognize potential anti-SARS-CoV-2 M^pro^ molecules [17]. Understanding the interaction between tiny compounds, often known as receptors and ligands, is aided by computational methods. In determining the interaction affinity of the likely lead compound with the target protein, molecular docking and MD simulations are used. Molecular docking studies estimate the interaction affinity and binding energy involved in the interaction between a ligand and a receptor [17]. MD simulation simulates a molecule to understand the system's dynamic performance.

A successful technique for discovering new medicines with anti-viral activity against COVID 19 is the drug repurposing strategy, which can be used in various ways [18]. Generating a pharmacophore from several cocrystallized inhibitors is one of the finest approaches to uncovering new molecules with desired binding affinity to the viral M^pro^'s active site in modern virtual screening. This assists in investigating the fundamental qualities essential for an anti-SARS-CoV-2 M^pro^ molecule. Numerous valued studies on drug repurposing techniques have recently been conducted against SARS-CoV-2 [19–22]. However, no actual medicinal remedy to address the viral infection has been presented, necessitating a quick, strategic, and cost-effective drug discovery strategy, which might be achieved by using targeted in silico and virtual screening techniques.

This study's primary goal was to use ZINC database compounds to find molecules against the virus's M^pro^. The ZINC database is a curated collection of commercially available and annotated compounds. It provides 3D compounds in numerous formats compatible with most docking programs. Several researchers have used ZINC database to identify different compounds that possess inhibitory effects on various disease-causing bacteria or viruses. For instance, Pinto et al. [23] used the ZINC database to identify new anti-tuberculosis molecules. A combination of bioinformatics methods, hierarchical virtual screening, and MD simulation is employed to find a potent anti-SARS-CoV-2 M^pro^ molecule. A pharmacophore model is first created using already identified potent anti-viral M^pro^ compounds from virtual screening of known drugs.

Mengist et al. [3] discovered 15 potent anti-viral M^pro^ molecules using in silico methods, and the first five most potent inhibitors were dipyridamole, candesartan, cilexetil, hydroxychloroquine, and chloroquine. The authors also identified cinanserin, nelfinavir, baicalin, and baicalein as other SARS-CoV-2 M^pro^ inhibitors using in vitro/in vivo techniques [3]. The structures of these natural molecules (eight of them: candesartan cilexetil, chloroquine, dipyridamole, hydroxychloroquine, cinanserin, nelfinavir, baicalin, and baicalein) were retrieved from the PubChem database and used to develop a pharmacophore hypothesis and pharmacophore model. This pharmacophore was used to test the ZINC database of natural molecules, and the eventual hits were filtered via drug-likeness rules and criteria. The protein's interaction with the ligands was then studied via molecular docking, and the best protein-ligand complex was determined and its stability measured through MD simulation.

## 2. Materials and Methods

All the bioinformatics and computational studies were performed with Intel®core™ 2 Duo CPU E7600 @ 3.06 GHz processor alongside the various installed software package: PyMOL, PyRx, and GROMACS, and web servers and databases: PubChem, OPENBABEL, PharmaGist, ZINCPHARMER, and SwissADME. The several SARS-CoV-2 M^pro^ inhibitors that exist in current literature were preferred as ligands to develop a pharmacophore hypothesis and design a pharmacophore model because they bind to the active site of the virus's M^pro^. Some of the SARS-CoV-2 M^pro^ inhibitors identified through in vitro/in vivo techniques include cinanserin, nelfinavir, baicalin, and baicalein, while those discovered via in silico method comprise candesartan cilexetil, chloroquine, dipyridamole, and hydroxychloroquine [3]. These eight anti-SARS-CoV-2 M^pro^ compounds were used during pharmacophore modeling.

### 2.1. Retrieval of the Ligands' Structures

The 2D and 3D structures of the eight ligands of interest were retrieved from the PubChem library database (https://pubchem.ncbi.nlm.nih.gov/). In this case, the 3D structures of the eight compounds are crucial for identifying their common pharmacophore features.

### 2.2. Pharmacophore Designing/Modeling

The 3D structures of the eight compounds were retrieved from the PubChem library database in the .sdf format. Finding the common pharmacophore features of the eight compounds required the use of PharmaGist, a freely available web-based server (https://bioinfo3d.cs.tau.ac.il/PharmaGist/). PharmaGist works only with 3D structures in the .mol2 format. Therefore, the 3D structures of the eight compounds were converted from the .sdf to .mol2 format using OPENBABEL, another free web-based server (http://www.cheminfo.org/Chemistry/Cheminformatics/FormatConverter/index.html). The .mol2 formatted compounds were compressed into a zip file and submitted to PharmaGist to be aligned, and common pharmacophore features detected.

Similarly, OPENBABEL is another essential computational tool necessary when dealing with compounds of different formats. It is an open, collaborative chemical toolbox that allows people to search, convert, analyze, and store chemical data [24]. Its main role is to convert chemical data from one format to another, evident via its utilization in different studies that exist in current literature. For example, Álvarez-Carretero et al. [25] used OPENBABEL tools as part of their virtual screening package in their study. Version 2.3 of OPENBABEL has the capability of interconverting over 110 formats, making it a vital library with a wide variety of molecular and chemical data that implements a broad scope of cheminformatics algorithms, from aromaticity detection and partial charge assignment to canonicalization and bond order perception [26]. This broad array of capabilities enables the OPENBABEL library to function in tandem with programming languages such as Python to compute molecular descriptors for different compounds [27].

After generating a ligand-based 3D pharmacophore of medicinal compounds using the PharmaGist web server, suitable pharmacophores were chosen from among the many results produced based on the web server's scores. The pharmacophore with the highest score was chosen because it represents the highest structural conformation similarity of the eight molecules. Furthermore, the pharmacophore had to be based on the maximum molecules aligned in the pharmacophore design [28]. The features of the 3D pharmacophore were uploaded to PyMOL 2.5.2 for visualization and labeling of the different pharmacophore features.

### 2.3. Pharmacophore-Based Virtual Screening

The 3D pharmacophore was then loaded into the ZINCPHARMER web server (http://zincpharmer.csb.pitt.edu/pharmer.html) to locate active compounds that can inhibit the SARS-CoV-2 M^pro^ through a virtual screening process.

To refine the outcome obtained from ZINCPHARMER, various drug-likeness filters, integrated into the SwissADME web tool, including Lipinski's Rule, Ghose Filter, Veber Filter, Egan Filter, and Muegge Filter, were applied to select the compounds with desirable pharmacokinetic properties. The molecules that satisfied all the requirements of all the five drug-likeness filters were selected for molecular docking studies.

### 2.4. Target Protein 3D Structure Retrieval and Preparation

The target protein was SARS-CoV-2 M^pro^. Its 3D structure was retrieved from the Protein Data Bank (PDB) database (https://www.rcsb.org/) using PDB ID 6Y2E. The 3D structure of the SARS-CoV-2 M^pro^ was necessary for molecular docking to show its interaction with the different potential anti-viral candidates.

After the retrieval of SARS-CoV-2 M^pro^ stucture, it had to be prepared for molecular docking. BIOVIA Discovery Studio 2021 was used to prepare the 3D structure of the virus's enzyme. The target protein was loaded into the BIOVIA Discovery Studio 2021, and all water molecules and heteroatoms were removed because they are not involved in the binding of the ligand to the protein. Deleting them eases computations and prevents distortion of the pose search that would otherwise occur if water molecules and heteroatoms were not cleared from the binding pocket. Similarly, polar hydrogens were added to the 3D structure of the virus's main protease. Polar hydrogens assist in finding the hydrogen bond interactions and make it possible to determine the binding affinity of the ligand against the virus's main protease. After preparation, the 3D structure of SARS-CoV-2 M^pro^ was saved as a .pdb file.

### 2.5. Molecular Docking of Selected Drug Candidates with SARS-CoV-2 M^pro^

The ligands were docked with the target protein using Autodock Vina, which is embedded into the Pyrx software. The prepared target protein was first loaded into the Pyrx software and converted from .pdb to .pdbqt format. The selected ligand molecules were then loaded into the Pyrx software as well. The energy of all the ligands were then minimized. All the ligands were then converted to Autodock ligands (PDBQT format). Molecular docking was done, and the ligands with the lowest binding energies were selected as the final drug candidates after analysis using Autodock tools. The same docking process was performed using the target protein (SARS-CoV-2 M^pro^), and the eight ligands utilized to create the pharmacophore model (candesartan cilexetil, chloroquine, dipyridamole, hydroxychloroquine, cinanserin, nelfinavir, baicalin, and baicalein). The two sets of docking results, the eight ligands and the final leads, were compared. The *x*, y, and *z* coordinates of the grid that was utilized during docking were –16.5791, –25.7662, and 15.0336, respectively. The grid dimensions were 34.1315 Å (*x*), 64.0261 Å (*y*), and 61.7477 Å (*z*).

### 2.6. Pharmacokinetic Properties of the Final Drug Candidates

The SMILES for the final drug candidates were copy-pasted into the SwissADME web server, and their pharmacokinetic properties were examined. Their oral bioavailability was assessed and presented as bioavailability radars. Additionally, the Brain Or IntestinaL EstimateD permeation (BOILED-Egg) analysis was performed for all the final drug candidates.

### 2.7. Molecular Dynamics Simulation

GROMACS 2022 was used to perform molecular dynamics simulation on the docked complex of the final drug candidate, **ZINC000072307130,** to ascertain the docking results and undertake an in-depth assessment of the behavior of the final ligand within the binding pocket of the viral protein. CHARMM-GUI web server was used to generate the GROMACS MD files, topology file for the protein, and the topology and parameter files for the ligand, utilizing CHARMM36 force field. During the complex's solvation, the default waterbox size options were selected; the waterbox size was fit to protein size using a rectangular waterbox type with an edge distance of 10.0. Eighty-three K^+^ and 79 Cl^–^ ions were added to the complex using the Monte-Carlo ion placing method to neutralize the system and attain a physiological KCl concentration of 0.15 mM. Energy minimization was performed using the steepest descent for 5,000 steps. The minimized system was then equilibrated, subjected to a 100 ps run at a constant temperature of 300.00 K. This was followed by a 10 ns production run. The number of hydrogen bonds and the root mean square deviation (RMSD) were calculated. Microsoft Excel and VMD were used to generate the 2D RMSD plot and hydrogen bond plot, respectively. The principal component analysis (PCA) was also done. The eigenvalues and eigenvectors for the covariance matrices were diagonalized and solved to produce the principal components for the protein-ligand complex. The motion's amplitude and direction are shown, respectively, by the eigenvalues and eigenvectors. The GROMACS tool gmx covar was used to calculate the covariance matrix, construct, and diagonalize it. The GROMACS tool gmx anaeig was then applied to calculate overlaps between the trajectory coordinates and the computed principal components. The QtGrace software was then used to construct a scree plot PCA and a 2D projection of the trajectory.

## 3. Results

### 3.1. Retrieval of the Ligands' Structures

**Figure 1:**
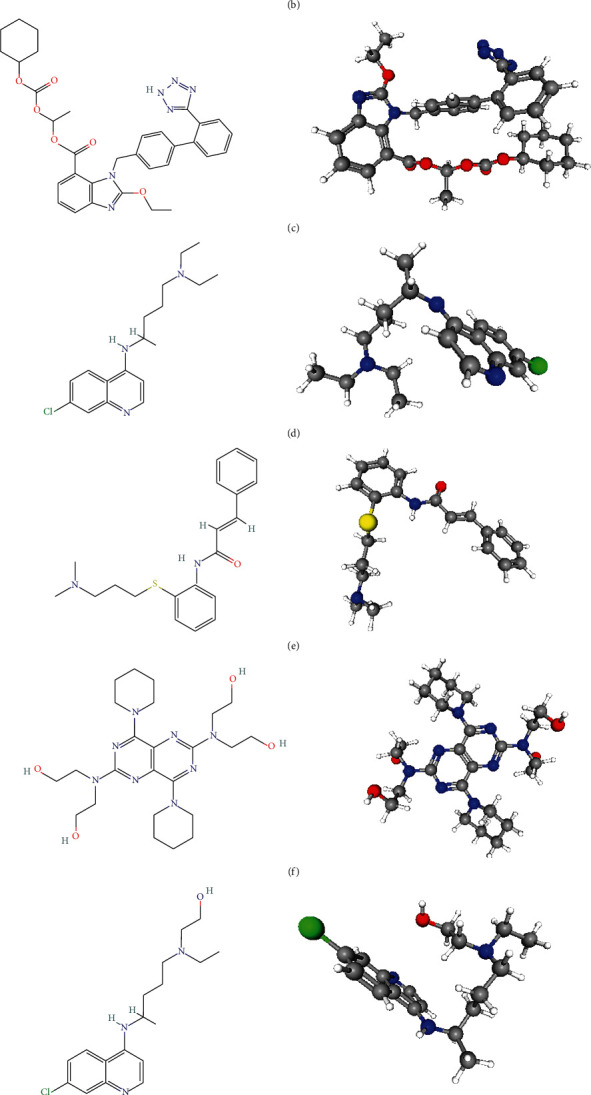
2D and 3D Structures of SARS-CoV-2 Inhibitors: (a) baicalein, (b) baicalin, (c) candesartan cilexetil, (d) chloroquine, (e) cinanserin, (f) dipyridamole, (g) hydroxychloroquine, and (h) nelfinavir.

From the PubChem library database, information on the compounds' PubChem CID, molecular formula, molecular weight, and 2D and 3D structures were collected. This information is summarized in Table 1 and Figure 1.

### 3.2. Pharmacophore Designing/Modeling

PharmaGist enabled the detection of the common pharmacophore features that the eight compounds share. The results are displayed in Tables 2 and 3.

**Figure 2:**
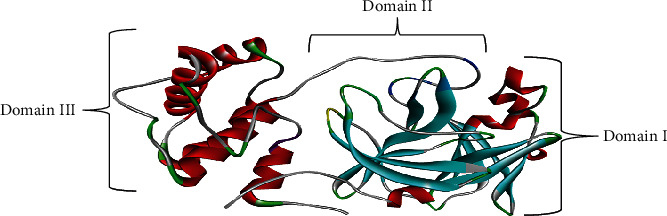
The pharmacophore with distances between its atoms. The pharmacophore has three features: one aromatic ring (AR), one hydrogen bond donor (DON), and one hydrogen acceptor (ACC). The distance between AR and DON is 1.7 Å. The distance between AR and ACC is 4.2 Å. The distance between ACC and DON is 3.4 Å.

From the PharmGist results above, the pharmacophore with the highest score of 15.875 under the section with 5 aligned molecules was chosen. This pharmacophore was visualized using PyMOL 2.5.2 as displayed in Figure 2. It had three pharmacophore features: hydrogen bond donor (HBD), hydrogen bond acceptor (HBA), and an aromatic ring (AR). The distances between the three atoms were also measured. The distance between HBA and HBD was 3.4 Å. The distance between HBA and AR was 4.2 Å. The distance between AR and HBD was 1.7 Å.

### 3.3. Pharmacophore-Based Virtual Screening

The virtual screening process performed via the ZINCPHARMER web server resulted in 18,009,471 hits. The simplified molecular-input line-entry system (SMILES) for the 28 molecules with RMSD values of 0.145 or lower were copy-pasted into the ZINC15 database (https://zinc15.docking.org/substances/home/), each SMILES per line. The ZINC15 database generated a .sdf file containing the 3D coordinates of 34 molecules. The additional molecules were the annotations of some of the 28 molecules retrieved from the ZINCPHARMER. These 34 molecules were subjected to a drug-likeness test. The SwissADME's inbuilt drug-likeness filters such as Lipinski, Ghose, Veber, Egan, and Muegge produced the results summarized in Table 4.

### 3.4. Target Protein 3D Structure Retrieval and Preparation

**Figure 3:**
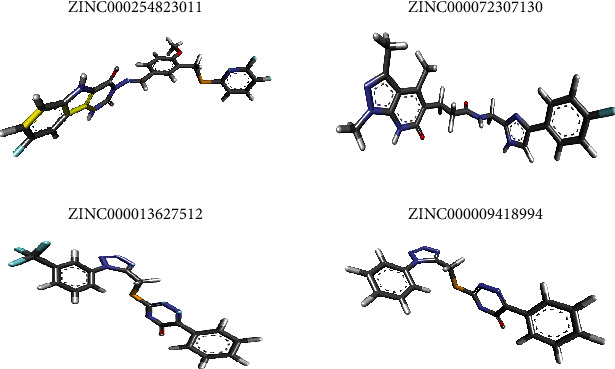
The 3D structure of prepared SARS-CoV-2 M^pro^. All the water molecules and other heteroatoms have been removed from SARS-COV-2 M^pro^ retrieved from PDB, ID 6Y2E. The three domains (I, II, and III) are indicated.

The 3D structure of the prepared SARS-CoV-2 M^pro^ is presented in Figure 3. It represents a homodimer with two protomers each comprising three domains (I, II, and III).

### 3.5. Molecular Docking of Selected Drug Candidates with SARS-CoV-2 M^pro^

**Figure 4:**
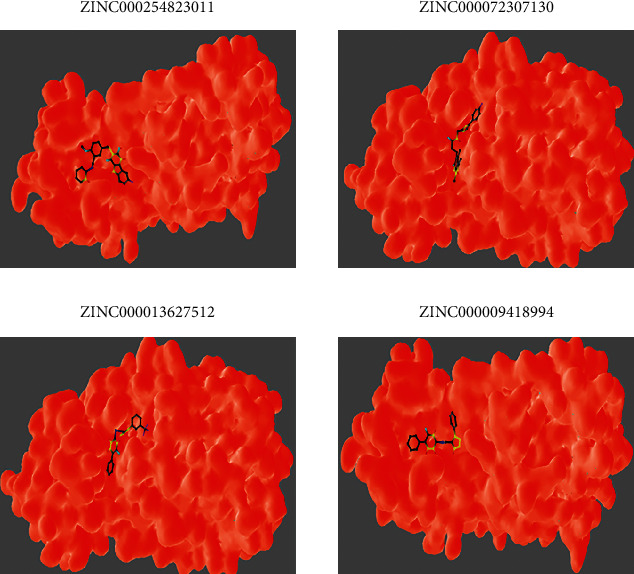
Image showing the 3D structures of the four final drug candidates.

**Figure 5:**
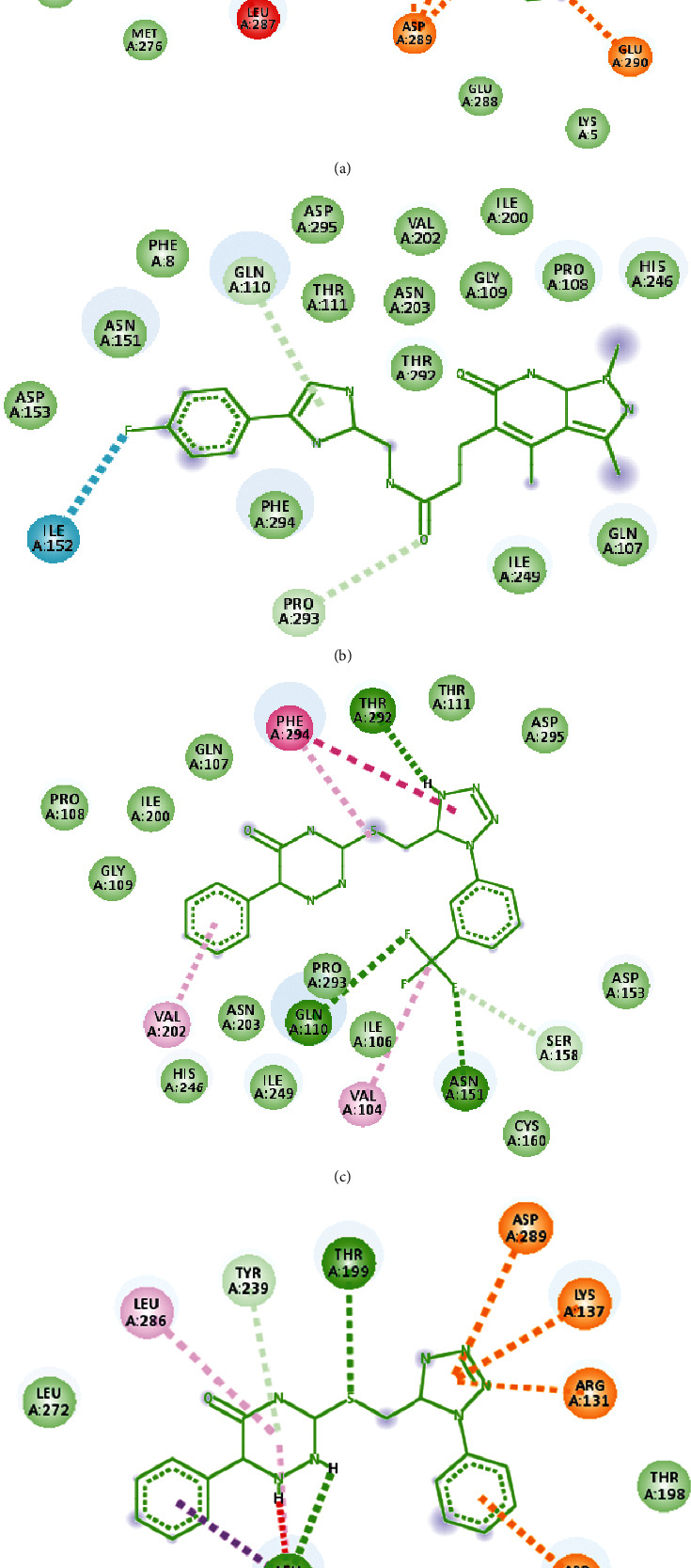
Molecular surface representation showing the four final drug candidates lying within the binding pockets of the target protein (PDB ID 6Y2E).

**Figure 6:**
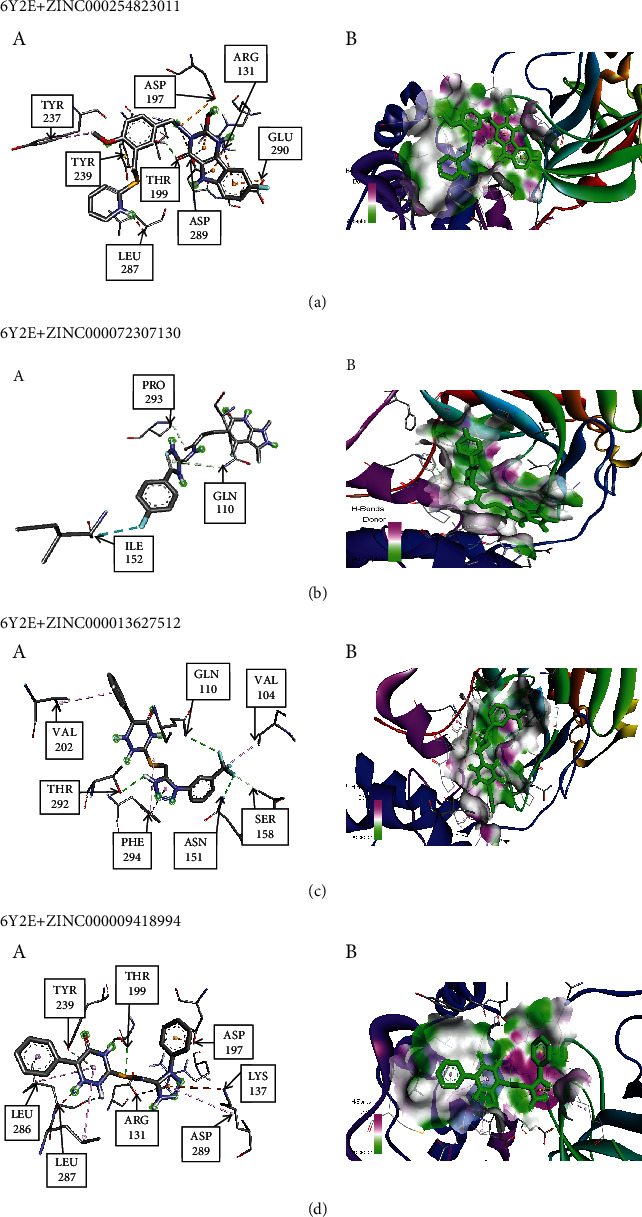
The 2D interaction diagrams showing the binding site residues. (a) 6Y2E+ZINC000254823011, (b) 6Y2E+ZINC000072307130, (c) 6Y2E+ZINC000013627512 and (d) 6Y2E+ZINC000009418994.

**Figure 7:**
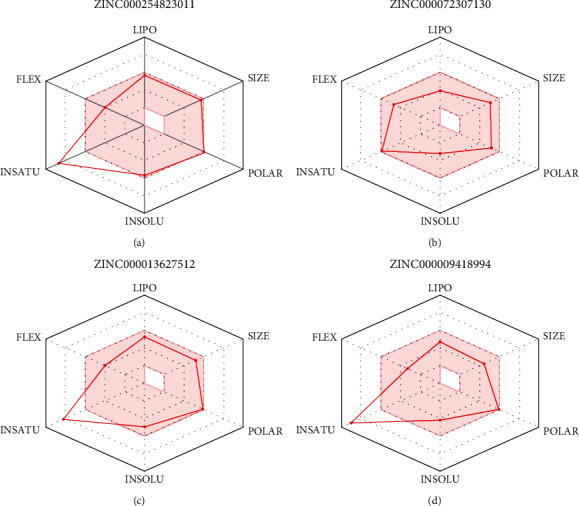
2D and 3D interaction diagrams showing the binding sites residues. (A) 3D structure of the four drug candidates showing their binding interaction with the target protein (SARS-CoV-2 Mpro. (B) 3D structure displaying the four drug candidates within the binding pocket of the target protein.

After examining the molecular docking complexes of all the 16 screened molecules with SARS-CoV-2 M^pro^, their binding energies were recorded. Four of the 16 ZINC molecules had the desirable lowest energies: **ZINC000009418994** (–8.2 kcal/mol), **ZINC000013627512** (–8.2 kcal/mol), **ZINC000072307130** (–8.5 kcal/mol), and **ZINC000254823011** (–8.6 kcal/mol). The lower the binding energy, the more stable the interaction because of higher binding affinity [28]. The binding energies of these four leads were compared with those of the eight ligands: candesartan cilexetil (–8.3 kcal/mol), chloroquine (–6 kcal/mol), dipyridamole (–6.4 kcal/mol), hydroxychloroquine (–6.4 kcal/mol), cinanserin (–6.5 kcal/mol), nelfinavir (–7.9 kcal/mol), baicalin (–8.4 kcal/mol), and baicalein (–7.5 kcal/mol). **ZINC000072307130** (–8.5 kcal/mol) and **ZINC000254823011** (–8.6 kcal/mol) had more desirable binding energies than all the eight ligands. **ZINC000009418994** (–8.2 kcal/mol) and **ZINC000013627512** (–8.2 kcal/mol) had preferred binding energies to those of six ligands used in developing the pharmacophore model. Therefore, **ZINC000072307130** and **ZINC000254823011** represent better options as drug candidates because they give anticipated interaction with SARS-CoV-2 M^pro^. The 3D coordinates of the 4 ZINC molecules were retrieved from the ZINC15 database and visualized in BIOVIA Discovery Studio 2021, as displayed in Figure 4. The molecular docking interactions of these ZINC molecules with the target protein are displayed in Figure 5. Further analysis of the docked protein-ligand complexes analysis was done using BIOVIA Discovery Studio 2021. The binding site residues for the interaction of each of the four drug candidates were identified. The 2D (Figure 6) and 3D (Figure 7) interaction diagrams showing the binding site residues are presented.

### 3.6. Pharmacokinetic Properties of the Final Drug Candidates

**Figure 8:**
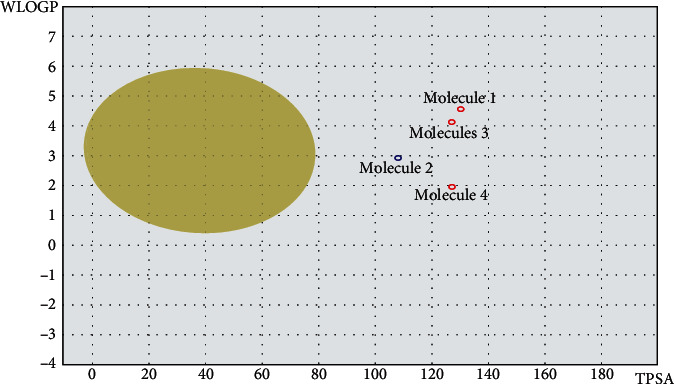
Image showing the bioavailability radars of the 4 final drug candidates. ZINC000254823011, ZINC000013627512, and ZINC000009418994 are not orally bioavailable because their red line radars do not lie within the pink region. ZINC000072307130 is orally bioavailable; its red line radar falls within the pink region.

The bioavailability radars of the four final drug candidates are shown in Figure 8. From the radar plot, it is evident that only **ZINC000072307130** might be orally bioavailable because its physicochemical properties, denoted by the red line radar, lie within the pink region, which is the optimal zone. However, the other three potential drug candidates, **ZINC000254823011**, **ZINC000013627512**, and **ZINC000009418994** show low saturation (Fraction Csp3) values of 0.08, 0.11, and 0.06, respectively, making them unsuitable for oral bioavailability.

**Figure 9:**
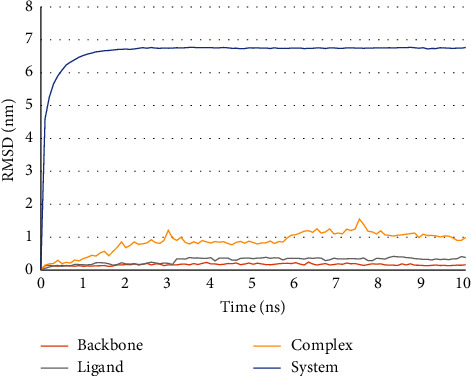
Boiled egg prediction of blood brain barrier permeability and gastrointestinal absorption for the 4 final drug candidates. Molecule 2 is a P-glycoprotein (P-gp) substrate, indicated by the blue dot, pointing out its ease of excretion from the body. Molecules 1, 3, and 4 are not P-glycoprotein (P-gp) substrates, exhibited by the red dots, signifying that they might not be easily excreted from the body.

The BOILED-Egg analysis was presented in Figure 9. The blue dot in the egg white region shows molecule 2 (**ZINC000072307130**) as a P-glycoprotein (P-gp) substrate, while the red dots indicate that the other three drug candidates (**ZINC000254823011**, **ZINC000013627512**, and **ZINC000009418994**) possess structural obstacles that bar them from binding to P-gp, thus creating drug excretion problems that trigger other toxicity outcomes. Therefore, from the pharmacokinetic properties analysis for the four drug candidates, **ZINC000072307130** stands out as the molecule with desirable drug characteristics.

### 3.7. Molecular Dynamics Simulations

**Figure 10:**
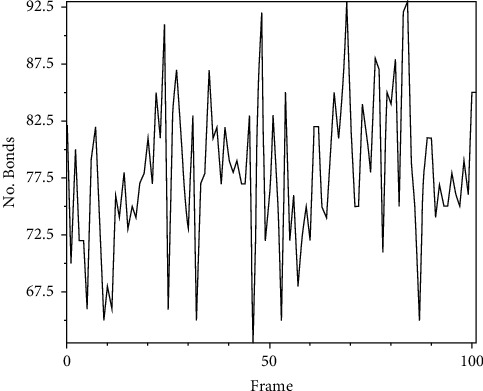
Assessment of RMSD plot during 10ns MD simulation. The SRAS-CoV-2 Mpro backbone has been represented in red, the ligand ZINC000072307130 in gray, the complex in yellow, and the entire system in blue. The RMSD plot ascertains the stability of the ligand, viral protein, and complex because of the extremely low RMSD values and insignificant structural fluctuations.

The RMSD plot (Figure 10) shows the trajectories of the analyzed complex and the entire system attain equilibrium after approximately 2 ns and remain steady (without major fluctuations) through the remaining 8 ns simulation. The ligand (ZINC000072307130) and the viral protein appear stable without significant structural fluctuations throughout the 10 ns simulation period.

**Figure 11:**
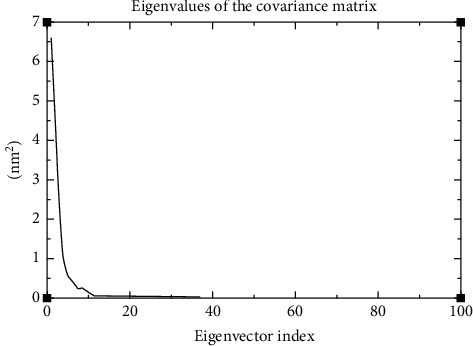
The number of hydrogen bonds calculated over 10ns MD simulation.

The hydrogen bonds plot ascertains the stability of the complex formed between the ligand and the viral protein. The number of hydrogen bonds formed between the viral protein and the ligand over the period of the MD simulation was majorly between 65 and 95 (Figure 11). This extensive hydrogen bond network that the ligand forms with the viral protein indicates its capability to act as an inhibitory molecule against SARS-CoV-2.

**Figure 12:**
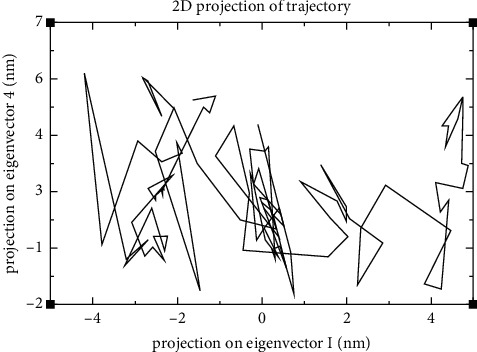
Scree plot for PCA on the MD coordinate data of the protein-ligand complex. The plot shows the variation extent each PC captures from the data. The eigenvalues (nm2) stand for the variation amount while the eigenvector index represents the PCs.

**Figure 13:**
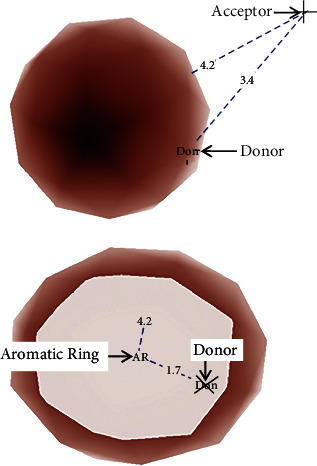
2D projection of trajectory of four eigenvectors or PCs. The PCs define the relative contribution of x, y, and z component of each c-alpha atom to the interrelated mode of motion conforming to that component.

To display the collective motion of the protein and ligand in the generated complex, PCA of MD simulation trajectories was conducted. Figures 12 and 13 show the outcomes of the PCA analysis of the test system. The resultant PC analysis scree plot, which displays the variance versus its eigenvector index, is shown in Figure 12. These findings show that the eigenvalues of the first three principal components (PC) are greater than 1. These three factors account for a sizable portion of the data's variation. The scree plot demonstrates that after the fourth PC, the eigenvalues begin to form a straight line. Therefore, the first four PCs are used to generate the 2D projection of the trajectory plot (Figure 13). Results demonstrated that the binding of the ligand considerably reduced the collective motion of the SARS-CoV-2 M^pro^.

## 4. Discussion

Comprehending the virus-receptor interaction mechanism responsible for COVID 19 progression can inform the production of a suitable chemotherapeutic intervention against the viral protein. Currently, there exists no medication or anti-viral treatment against SARS-CoV-2. The WHO has proactively announced COVID 19 infection as a worldwide pandemic. Finding natural molecules with anti-viral activity against SARS-CoV-2 can be the quickest way of developing therapeutic agents against COVID 19 disease. Such molecules can be directly tested as anti-SARS-CoV-2 drugs and processed for COVID 19 trials. The current study explored in silico means to find SARS-CoV-2 M^pro^ inhibitors as potential drug candidates against COVID 19.

All the methods and processes were performed with Intel®core™ 2 Duo CPU E7600 @ 3.06 GHz processor alongside the various installed software packages: PyMOL, PyRx, and GROMACS and web servers and databases: PubChem, OPENBABEL, PharmaGist, ZINCPHARMER, and SwissADME. This methodology is replicable using computers of different specifications. Previous studies by Jayaraj et al. [16] reported the use of these specifications.

Basic information (Table 1) and 2D and 3D structures (Figure 1) of the eight SARS-CoV-2 M^pro^ inhibitors that exist in current literature: cinanserin, nelfinavir, baicalin, baicalein, candesartan cilexetil, chloroquine, dipyridamole, and hydroxychloroquine, were retrieved from the PubChem library database. As a public chemical database, PubChem serves the scientific communities and the general public as well. Kim [29] acknowledges the integral role of the PubChem library database by pointing out how it allows users to quickly retrieve a list of records annotated with specific categorization or ontological terms. It supports various types of structure searches, including superstructure, substructure, 2D and 3D similarity, and identity searches [29].

Since these eight molecules inhibit SARS-CoV-2 M^pro^ by binding to its active site, they can be used to develop a pharmacophore model utilized for screening for other anti-SARS-CoV-2 M^pro^ molecules in different databases, in this case, the ZINC database. The basic information of the eight inhibitors is presented for easy and quick retrieval of those particular compounds in future research. The 2D and 3D structures of the inhibitors are necessary for structural comparison (providing additional structural information of the compounds) and pharmacophore modeling and subsequent usage in PharmaGist, virtual screening, and docking, respectively. Pinto et al. [23] used a similar approach to identify anti-tuberculosis molecules from natural sources. After presenting 2D and 3D structures of a compound exhibiting biological activity to their target, they used six molecules as the training set to construct pharmacophore models, choosing one with the best GALAHAD^TM^ parameters [23].

In this study, the eight SARS-CoV-2 M^pro^ inhibitors were the training set aiding the construction of a pharmacophore model. PharmaGist was a suitable pharmacophore model constructing tool because it aligns the eight molecules and detects their common pharmacophore features. It is a web server that aids in detecting ligand-based pharmacophores. The input is a 3D representation of a set of drug-like ligands. The result is a list of pharmacophore candidates. These are three-dimensional patterns of physicochemical properties shared by all or some of the input ligands. In addition, the output gives a 3D superposition of conformations of input ligands that share it for each potential pharmacophore. PharmaGist advises that the challenge be solved by several flexible alignments of drug-like molecules (Schneidman-Duhovny et al., 2008). Several scholars have used PharmaGist to derive the 3D pharmacophore in their studies.

For instance, Ferreira et al. [30], Raphael and Shanmughan [31], and Zainab et al. [28] used this web server in their respective studies to design a pharmacophore model that they subsequently used in ZINC databases to identify potential hit compounds. However, PharmaGist requires the input files to be in .mol2 format. Therefore, the .sdf formats of the eight molecules retrieved from the PubChem library database had to be converted to .mol2 format using OPENBABEL. OPENBABEL is another essential computational tool necessary when dealing with compounds of different formats. It is an open, collaborative chemical toolbox that allows people to search, convert, analyze, and store chemical data [24]. Its main role is to convert chemical data from one format to another, evident via its utilization in different studies that exist in current literature. For example, Álvarez-Carretero et al. [25] used OPENBABEL tools as part of their virtual screening package in their study. Version 2.3 of OPENBABEL has the capability of interconverting over 110 formats, making it a vital library with a wide variety of molecular and chemical data that implements a broad scope of cheminformatics algorithms, from aromaticity detection and partial charge assignment to canonicalization and bond order perception [26]. This broad array of capabilities enables the OPENBABEL library to function in tandem with programming languages such as Python to compute molecular descriptors for different compounds [27].

The PharmaGist results (Tables 2 and 3) present the input molecules and a list of pharmacophore candidates, respectively. Table 2 shows the eight SARS-CoV-2 inhibitors with their respective number of atoms, pharmacophore features, and spatial features. The pharmacophore features of interest included the number of aromatic rings, hydrophobic groups, hydrogen bond donors, hydrogen bond acceptors, cations, and anions. The list of pharmacophore candidates is displayed in Table 3. These are three-dimensional patterns of physicochemical properties shared by all or some of the input ligands. In addition, the output gives a 3D superposition of conformations of input ligands that share it for each potential pharmacophore. The pharmacophore with the highest score (15.875) was preferred because it represents the highest structural conformation similarity of the eight molecules. It had to be based on the maximum number of molecules aligned in the pharmacophore design [28]. The greater the number of molecules aligned, the better the results obtained in terms of the common pharmacophore features the eight inhibitors share. Scholars like Ferreira et al. [30], Raphael and Shanmughan [31], Ravindran et al. [32], and Zainab et al. [28] have used PharmaGist in their respective studies to develop pharmacophore models. Therefore, it is a suitable in silico tool to develop pharmacophores for virtual screening. The eight inhibitors had three common pharmacophore features (Figure 2). They all possessed at least one aromatic ring, one hydrogen bond acceptor, and one hydrogen bond donor.

The visualization and labeling of the pharmacophore model using PyMOL 2.5.2 identified the different pharmacophore features. PyMOL is an open-source computational tool that can assist in visualizing a pharmacophore model. It enables the labeling of different atoms in a pharmacophore model and measuring the distances between those particular atoms. The three pharmacophore features of the pharmacophore model were labeled as aromatic ring (AR), hydrogen bond acceptor (ACC), and hydrogen bond donor (DON)(Figure 2 ). The distance between ACC and DON was 3.4 Å. The distance between ACC and AR was 4.2 Å. The distance between AR and DON was 1.7 Å (Figure 2). This schematic representation of the pharmacophore model helped during virtual screening to identify natural molecules in the ZINC database that have at least one AR, one ACC, and one DON with approximately the same distances between them. When preparing to perform molecular docking in their studies, Ferreira et al. [30] and Ravindran et al. [32] also used different versions of PyMOL to generate and visualize their pharmacophore models. The pharmacophore model was essential during visual screening to identify structurally similar compounds.

Pharmacophore-based virtual screening via the ZINCPHARMER web server identified potential drug candidates against SARS-CoV-2 M^pro^. Before wet-lab experiments, one of the standard procedures in drug development is virtual screening. This procedure involves calculating the drug candidate's binding affinity for a target protein. During the interaction, virtual screening is also performed to determine possible binding modalities of the drug candidate and other drug-like small molecules to the target protein [28]. Using high-performance computing (HPC) infrastructure tools, the most notable drug candidates with promising binding affinity for the target protein may be filtered out (Schneidman-Duhovny et al., 2008). Different bioactive compounds that can interact with the target protein can be identified using the virtual screening approach. ZINCPHARMER is one of the web-based platforms for virtual screening for the pharmacophore against the ZINC drug database. Molecules with low root mean square deviation (RMSD) values from the active sites of pharmacophores were chosen for docking studies from the various hit compounds displayed by the ZINC database [7]. Compounds with low RMSD values are preferred because they are highly structurally similar to the pharmacophore's active sites.

The ZINCPHARMER web server recognized the atoms and the distances between them and searched for molecules with the same structural conformation. This process resulted in 18,009,471 hits. Molecules with the lowest RMSD values, 0.145 or lower, were chosen for docking studies. The lower the RMSD value of a molecule, the lower its structural deviation from the pharmacophore's active sites [7]. Therefore, low RMSD values denote a molecule's high structural similarity to the pharmacophore's active sites. The total number of molecules that were selected was 28. Such molecules are believed to have the capability of binding to SARS-CoV-2 M^pro^ and preventing it from facilitating COVID 19 progression. Therefore, based on their binding affinities to SARS-CoV-2 M^pro^, they can be selected as potential drug candidates against SARS-CoV-2 M^pro^. Several scholars have also used ZINCPHARMER as their preferred pharmacophore-based virtual screening web server while undertaking their respective studies [28, 30, 33–35].

From the ZINCPHARMER web server, the SMILES of each of the 28 molecules were essential in generating their 3D coordinates in the .sdf format from the ZINC15 database, necessary for docking studies. The ZINC15 database generated 3D coordinates of 34 molecules from the SMILES of 28 molecules, signifying that some of the additional molecules share SMILES with some of the 28 molecules. Therefore, the additional molecules can be considered structural analogs or annotations of some of the 28 molecules retrieved from the ZINCPHARMER web server. The ZINC15 database is a crucial computational tool when filtering specific molecules from a pool of different drug-like compounds. Al-Aziz et al. [36], Susanti et al. [37], and Wu et al. [38] used ZINC15 to identify specific molecules of interest in their studies. Al-Aziz et al. [36] leveraged the ZINC15 database to filter out 1,282 FDA and in-clinical approved drugs from approximately 0.5 million protomers of large compounds. Wu et al. [38] recognized vital compounds of Huangqin decoction (HQD) on ulcerative colitis using the ZINC15 database. Susanti et al. [37] performed pharmacophore-based virtual screening of the ZINC15 database to identify CDK4/6 inhibitors. The molecules retrieved from the ZINC15 database were subjected to further filtering using drug-likeness filters in the SwissADME web server.

The SwissADME is a tool used to investigate the drug-likeness, physicochemical parameters, and pharmacokinetic properties of molecules [39]. It is a free virtual screening web tool used to evaluate medicinal chemistry, drug-likeness, and pharmacokinetics friendliness of small molecules (Daina et al., 2017). It has inbuilt drug-likeness filters that were used to determine which molecules among the 34 satisfied the different drug-likeness requirements. The drug-likeness filters that were used include Lipinski, Ghose, Veber, Egan, and Muegge. The results summarized in Table 4 show that 16 of the 34 molecules satisfied all the requirements of the 5 drug-likeness filters. These results signify the possibility of the 16 compounds being used as drug candidates against COVID 19. Geronikaki et al. [39] used SwissADME to examine the drugability of various bioactive compounds in their study. Fekadu et al. [40] also acknowledged the significance of SwissADME as an in silico tool by using it to assess the drug-like properties of different compounds in their study. Even with all the 16 molecules satisfying the requirements of the SwissADME drug-likeness filters, their success as drug candidates against COVID 19 depended on their binding affinities to SARS-CoV-2 M^pro^ as well; hence, they were docked to the viral main protease using Pyrx software.

Molecular docking required the preparation of the 3D structure of the target protein (SARS-CoV-2 M^pro^). The target protein is readily available in the Protein Data Bank (PDB) using the PDB ID 6Y2E. Initially, the PDB ID (6Y2E) of SARS-CoV-2 Mpro was looked up from existing literature. Swain et al. [41], in their study, had already identified and retrieved the 3D crystal structure of the virus's main protease from the PDB database using the ID 6Y2E. The authors discovered that the SARS-CoV-2 M^pro^ comprises 306 amino acids and it can be used for docking studies [41]. The target protein preparation is essential for docking, a process easily done using BIOVIA Discovery Studio 2021 by removing all water molecules and heteroatoms because they were not involved in the binding of the ligands to the protein. Deleting them eases computations and prevents distortion of the pose search that would otherwise occur if water molecules and heteroatoms were not cleared from the binding pocket. Similarly, polar hydrogens were added to the 3D structure of the virus's main protease [28]. Polar hydrogens assist in finding the hydrogen bond interactions and making it possible to determine the binding affinity of the ligand against the virus's main protease. Zainab et al. [28] acknowledge the need to prepare a target protein by removing the heteroatoms and adding polar hydrogens in their study. The 3D structure of the prepared SARS-CoV-2 M^pro^ (Figure 3) was saved as a .pdb file. Some scholars have also used different versions of Discovery Studio as part of their computational tools to undertake various virtual screening and molecular docking processes. For instance, Chou et al. [42] and Rajpoot et al. [43] used Discovery Studio to prepare their target proteins before undertaking further virtual screening processes and molecular docking.

Autodock Vina, which is embedded in the Pyrx software, is a top-notch computational tool for molecular docking. For docking, the prepared SARS-CoV-2 M^pro^ was required in the .pdbqt format; hence, its conversion from the .pdb format. The ligands' preparation before docking was also necessary, involving their energies being minimized and their file formats converted to the .pdbqt format. During molecular docking, the *x*, y, and *z* grid coordinates were set as –16.5791, –25.7662, and 15.0336, respectively, and the grid dimensions as 34.1315 Å (*x*), 64.0261 Å (*y*), and 61.7477 Å (*z*). The docking results showed the binding affinities of the docked ligands with the target protein ranging from –7.0 kcal/mol to –8.6 kcal/mol. The molecules with binding affinities from –8.0 kcal/mol to –8.6 kcal/mol were selected for pharmacokinetic properties analysis. The lower the binding affinity, the stable the interaction between a ligand and its target protein because the ligand will have a higher binding affinity to its target protein [28]. Four of the 16 molecules fell within the desired binding affinity range: **ZINC000009418994** (–8.2 kcal/mol), **ZINC000013627512** (–8.2 kcal/mol), **ZINC000072307130** (–8.5 kcal/mol), and **ZINC000254823011** (–8.6 kcal/mol).

These 4 ZINC molecules represent better options as drug candidates because they give anticipated interaction with SARS-CoV-2 M^pro^. Therefore, their identities were sought via the PubChem library database. Currently, all four ZINC molecules are unknown. The 3D structures of the four molecules, as evident in Figure 4, were retrieved from the ZINC15 database to gain some insights into the structural conformations of the molecules. Since these molecules had already been docked to the target protein, the molecular docking interactions of these ZINC molecules with the target protein are displayed in Figure 5. The 2D (Figure 6) and 3D (Figure 7) structures of the protein-ligand complexes were analyzed using the BIOVIA Discovery Studio 2021. The structures show how the ligand binds to SARS-CoV-2 M^pro^ and even identifies the residues involved in the interaction between the ligand and the target protein. Even though these four ZINC molecules had desirable binding affinities to SARS-CoV-2 M^pro^, with **ZINC000254823011** having the best binding affinity score, as drug candidates against COVID 19, their pharmacokinetic properties were assessed to determine their oral bioavailability and permeation capabilities.

SwissADME was used to analyze the pharmacokinetic properties of the four final drug candidates. Based on the oral bioavailability radars and the BOILED-Egg outcome, Figures 8 and 9, respectively, **ZINC000072307130** exhibited oral bioavailability and permeation properties. However, the other three molecules, **ZINC000254823011**, **ZINC000013627512**, and **ZINC000009418994,** did not exhibit oral bioavailability and permeation properties. These results prove that **ZINC000072307130** is a better drug candidate than the other three molecules. Its saturation value (Fraction Csp3) of 0.27 is higher than the required threshold of 0.25, indicating its desirable high saturation. On the other hand, the other three ZINC molecules, **ZINC000254823011**, **ZINC000013627512,** and **ZINC000009418994** have low saturation values of 0.08, 0.11, and 0.06, respectively, making them unsuitable for oral bioavailability. Figure 9 shows molecule 2, which is **ZINC000072307130**, denoted by a blue dot within the egg-white region. It means that the molecule can act as a P-gp substrate, creating ease in its excretion. The other three molecules are denoted by red dots that signify the existence of structural barriers that can prevent them from binding to P-gp, creating drug excretion problems that trigger toxicity outcomes. In this regard, **ZINC000072307130**, which has a binding affinity score of –8.5, suitable oral bioavailability, and desired permeation capability, is the best drug candidate against COVID 19. Even though the binding affinity score of **ZINC000254823011** is lower than that of **ZINC000072307130**, the molecule might not function well as a drug against COVID 19 because of its poor oral bioavailability and permeation. Nevertheless, it can still be modified through in silico approaches and used in future in vitro research for COVID 19 drug development.

MD simulation was performed to assess the stability of the protein-ligand complex formed between **ZINC000072307130** and the target protein, SARS-CoV-2 M^pro^. The RMSD values of the SARS-CoV-2 Mpro-ZINC000072307130 complex over a period of 10 ns were used to monitor the stability of the docked complex of ZINC000072307130 with the viral protein. From Figure 10, it is clear that the complex is stable because of the low RMSD values and insignificant structural fluctuations. The hydrogen bond plot (Figure 11) also shows that the ligand can be used as an inhibitor against SARS-CoV-2 M^pro^ because of the high number of hydrogen bonds in the protein-ligand complex. Several scholars have also used 10 ns MD simulation in similar studies. For example, Poli et al. (2022) performed a 10 ns MD simulation in their study aimed at identifying novel PIN1 inhibitors. Similarly, Chunduru et al. (2021) performed a 10 ns MD simulation in their study that focused on assessing the anti-viral activity of new molecules against 3C-like protease of novel SARS-CoV and COVID 19.

A common analytical approach for evaluating the dimensionality reduction of huge datasets is the PCA. Additionally, it is a method that is frequently used in MD simulations to depict the slow, functional motions of biomolecules [44, 45]. The PCA results indicate the relative compactness of the protein structure after binding the lead molecule at the active site (Figure 12). They display the main motions contained in the MD trajectory, particularly within the first four eigenvectors' principal components before the variations within the system reduce significantly (Figure 13). The results show that even with a disturbance to the system and its relative motion, its steady state is reached, making it a stable system. This further proves the stability of the protein-ligand complex and the suitability of the lead compound as a potential COVID 19 drug.

## 5. Conclusion

The worldwide challenge in the form of the COVID 19 pandemic has inspired researchers to find, discover, and repurpose the existing and well-characterized natural molecules as possible inhibitors of SARS-CoV-2. SARS-CoV-2 has several structural and nonstructural proteins that act as targets of various anti-viral compounds and drugs. The main protease, one of the structural proteins of the virus, is one of the essential targets. Several researchers are keen on SARS-CoV-2 M^pro^ because blocking its role in COVID 19 progression can reduce the spread of the virus. The present study documents viral main protease as the target protein. Through pharmacophore-based virtual screening for natural compounds from the ZINC database, SARS-CoV-2 M^pro^ inhibitors were identified. ZINC database is a curated collection of commercially available molecules prepared particularly for virtual screening purposes.

The hierarchical virtual screening workflow was carried out for SARS-CoV-2 M^pro^. Overall, 16 molecules were docked with SARS-CoV-2 M^pro^. Four molecules with the lowest binding energies to the target protein, ZINC000254823011, ZINC000072307130, ZINC000013627512, and ZINC000009418994, were selected as the final drug candidates against SARS-CoV-2. Having the lowest binding energies to SARS-CoV-2 M^pro^ shows that the four drug candidates had the highest binding affinities to the target protein. Therefore, they have the best inhibitory function against the main protease of the viral protein. Further pharmacokinetics analysis of the four final drug candidates revealed that ZINC000072307130 was the only orally bioavailable molecule with desirable pharmacokinetic properties. MD simulation performed proved that the SARS-CoV-2 M^pro^-ZINC000072307130 complex is stable, making the ZINC molecule a probable SARS-CoV-2 M^pro^ inhibitor.

### 5.1. Recommendations

This study gives great promise in drug discovery research against COVID 19. The outcomes present novel opportunities for additional investigation of the active constituents of natural compounds in the ZINC database as well as other similar databases for an effective treatment procedure to battle SARS-CoV-2 and other microbial illnesses with negligible side effects. Future research can focus on applying this molecule in omicron variants and affirming its conformational alterations and binding stability, as well as assessing its inhibitory capability in vivo and in vitro against SARS-CoV-2.

## Figures and Tables

**Table 1:** Basic information on the anti-SARS-CoV-2 M^pro^ compounds retrieved from the PubChem library database.

Molecule	Name	PubChem CID	Molecular formula (MF)	Molecular weight (MW)
1	Baicalein	5281605	C_15_H_10_O_5_	270.24
2	Baicalin	64982	C_21_H_18_O_11_	446.4
3	Candesartan cilexetil	2540	C_33_H_34_N_6_O_6_	610.7
4	Chloroquine	2719	C_18_H_26_ClN_3_	319.9
5	Cinanserin	5475158	C_20_H_24_N_2_OS	340.5
6	Dipyridamole	3108	C_24_H_40_N_8_O_4_	504.6
7	Hydroxychloroquine	3652	C_18_H_26_ClN_3_O	335.9
8	Nelfinavir	64143	C_32_H_45_N_3_O_4_S	567.8

**Table 2:** Input molecules with their detected features visualized.

	Molecule	Atoms	Features	Spatial features	Aromatic	Hydrophobic	Donors	Acceptors	Negatives	Positives
1	Cinanserin.mol2	47	9	8	2	3	2	2	0	0
2	Dipyridamole.mol2	72	20	14	2	4	6	6	0	2
3	Candesartancilexetil.mol2	78	21	20	5	3	3	9	0	1
4	Hydroxychloroquine.mol2	43	9	8	2	3	2	2	0	0
5	Baicalein.mol2	30	11	8	3	0	3	5	0	0
6	Baicalin.mol2	49	20	15	3	0	5	11	1	0
7	Nelfinavir.mol2	71	20	18	3	8	3	6	0	0
8	Chloroquine.mol2	42	8	8	2	4	0	2	0	0

**Table 3:** Aligned molecules with their common pharmacophore features sorted by score. The best pharmacophore model is based on the highest number of aligned molecules: 5 with a score of 15.875.

Score	Features	Spatial features	Aromatic	Hydrophobic	Donors	Acceptors	Negatives	Positive	Molecules
*Number of aligned molecules: 5*									
15.875	3	3	1	0	1	1	0	0	Nelfinavir.mol2 Cinanserin.mol2 Baicalein.mol2 Baicalin.mol2 Candesartancilexetil.mol2

*Number of aligned molecules: 4*									
15.875	3	3	1	0	1	1	0	0	Dipyridamole.mol2 Cinanserin.mol2 Baicalein.mol2 Baicalin.mol2
15.875	3	3	1	0	0	2	0	0	Dipyridamole.mol2 Baicalein.mol2 Baicalin.mol2 Chloroquine.mol2
15.875	3	3	1	0	2	0	0	0	Candesartancilexetil.mol2 Cinanserin.mol2 Baicalein.mol2 Baicalin.mol2
15.156	4	3	1	0	1	2	0	0	Baicalin.mol2 Cinanserin.mol2 Baicalein.mol2 Dipyridamole.mol2
14.697	3	3	1	0	0	2	0	0	Nelfinavir.mol2 Baicalein.mol2 Baicalin.mol2 Candesartancilexetil.mol2

*Number of aligned molecules: 3*									
25.720	5	5	2	0	2	1	0	0	Candesartancilexetil.mol2 Baicalein.mol2 Baicalin.mol2
25.720	5	5	2	0	2	1	0	0	Candesartancilexetil.mol2 Baicalein.mol2 Baicalin.mol2
25.720	5	5	2	0	1	2	0	0	Dipyridamole.mol2 Baicalein.mol2 Baicalin.mol2
22.505	5	4	2	0	1	2	0	0	Dipyridamole.mol2 Baicalein.mol2 Baicalin.mol2
22.045	4	4	2	0	1	1	0	0	Candesartancilexetil.mol2 Baicalein.mol2 Baicalin.mol2
22.045	4	4	2	0	0	2	0	0	Chloroquine.mol2 Baicalein.mol2 Baicalin.mol2
22.045	4	4	2	0	0	2	0	0	Candesartancilexetil.mol2 Baicalein.mol2 Baicalin.mol2
22.045	4	4	2	0	0	2	0	0	Candesartancilexetil.mol2 Baicalein.mol2 Baicalin.mol2
18.371	3	3	2	0	1	0	0	0	Hydroxychloroquine.mol2 Baicalein.mol2 Baicalin.mol2
18.371	3	3	2	0	1	0	0	0	Candesartancilexetil.mol2 Baicalein.mol2 Baicalin.mol2

**Table 4:** Drug-likeness test results of the 34 molecule*s*.

No.	Molecule	IUPAC name	Lipinski	Ghose	Veber	Egan	Muegge	Suitable?
1	ZINC000003190091	2-(4-oxo-4H-1,3-benzothiazin-2-yl)-N-phenylacetamide	Yes	Yes	Yes	Yes	Yes	Yes
2	ZINC000072307130	N-[[5-(4-fluorophenyl)-1H-imidazole-2-yl]methyl]-3-(1,3,4-trimethyl-6-oxo-7H-pyrazolo[3,4-b]pyridin-5-yl)propanamide	Yes	Yes	Yes	Yes	Yes	Yes
3	ZINC000013568736	N-(1,3-benzodioxol-5-ylmethyl)-2-[(7-cyano-2,4-dioxo-1H-thieno[3,2-d]pyrimidin-6-yl)sulfanyl]acetamide	Yes	Yes	No	No	No	No
4	ZINC000071282864	2-(4,4-dimethyl-14-methylsulfanyl-16-oxo-5-oxa-8-thia-10,12,13,15-tetrazatetracyclo[7.7.0.02,7.011,15]hexadeca-1(9),2(7),10,13-tetraen-12-yl)acetamide	Yes	Yes	No	No	No	No
5	ZINC000001851882	8-chloro-3-[(Z)-[4-methoxy-3-(pyridin-2-ylsulfanylmethyl)phenyl]methylideneamino]-1,5-dihydropyrimido[5,4-b]indole-2,4-dione	Yes	No	Yes	Yes	Yes	No
6	ZINC000001851878	Not Available	Yes	No	Yes	Yes	Yes	No
7	ZINC000254397769	Not Available	Yes	No	Yes	Yes	Yes	No
8	ZINC000008657429	Not Available	Yes	No	Yes	Yes	Yes	No
9	ZINC000254823011	Not Available	Yes	Yes	Yes	Yes	Yes	Yes
10	ZINC000013569219	8-fluoro-3-[(Z)-[4-methoxy-3-(pyridin-2-ylsulfanylmethyl)phenyl]methylideneamino]-1,5-dihydropyrimido[5,4-b]indole-2,4-dione	Yes	No	Yes	Yes	Yes	No
11	ZINC000003253987	3-methyl-1-[2-[4-(4-methylphenyl)-1,3-thiazol-2-yl]ethyl]-4-(trifluoromethyl)-7H-pyrazolo[3,4-b]pyridin-6-one	Yes	No	Yes	Yes	Yes	No
12	ZINC000003215661	1-[2-[4-(4-methoxyphenyl)-1,3-thiazol-2-yl]ethyl]-3-methyl-4-(trifluoromethyl)-7H-pyrazolo[3,4-b]pyridin-6-one	Yes	Yes	Yes	Yes	Yes	Yes
13	ZINC000003216023	3-methyl-1-[2-(4-phenyl-1,3-thiazol-2-yl)ethyl]-4-(trifluoromethyl)-7H-pyrazolo[3,4-b]pyridin-6-one	Yes	Yes	Yes	Yes	Yes	Yes
14	ZINC000004987707	2-[(7-cyano-2,4-dioxo-1H-thieno[3,2-d]pyrimidin-6-yl)sulfanyl]-N-[(4-methoxyphenyl)methyl]acetamide	Yes	Yes	No	No	No	No
15	ZINC000074668320	Not Available	Yes	Yes	Yes	Yes	Yes	Yes
16	ZINC000014354393	Not Available	Yes	Yes	Yes	Yes	Yes	Yes
17	ZINC000071282864	2-(4,4-dimethyl-14-methylsulfanyl-16-oxo-5-oxa-8-thia-10,12,13,15-tetrazatetracyclo[7.7.0.02,7.011,15]hexadeca-1(9),2(7),10,13-tetraen-12-yl)acetamide	Yes	Yes	No	No	No	No
18	ZINC000004293776	[(2R,3S,4S)-3,4-diacetyloxy-4-(2,6-dioxo-3H-purin-9-yl)oxolan-2-yl]methyl acetate	Yes	No	No	No	No	No
19	ZINC000226348870	Not available	Yes	No	No	No	No	No
20	ZINC000004293780	[(2R,3 R,4S)-3,4-diacetyloxy-4-(2,6-dioxo-3H-purin-9-yl)oxolan-2-yl]methyl acetate	Yes	No	No	No	No	No
21	ZINC000226348864	[(2S,3S,4R)-3,4-diacetyloxy-4-(2,6-dioxo-3H-purin-9-yl)oxolan-2-yl]methyl acetate	Yes	No	No	No	No	No
22	ZINC000004293779	[(2S,3S,4S)-3,4-diacetyloxy-4-(2,6-dioxo-3H-purin-9-yl)oxolan-2-yl]methyl acetate	Yes	No	No	No	No	No
23	ZINC000013410783	[(2S,3 R,4R)-3,4-diacetyloxy-4-(2,6-dioxo-3H-purin-9-yl)oxolan-2-yl]methyl acetate	Yes	No	No	No	No	No
24	ZINC000013410785	Not available	Yes	No	No	No	No	No
25	ZINC000004293782	[(2S,3 R,4S)-3,4-diacetyloxy-4-(2,6-dioxo-3H-purin-9-yl)oxolan-2-yl]methyl acetate	Yes	No	No	No	No	No
26	ZINC000013627408	6-benzyl-3-[(1-phenyltetrazol-5-yl)methylsulfanyl]-4H-1,2,4-triazin-5-one	Yes	Yes	Yes	Yes	Yes	Yes
27	ZINC000009341012	6-benzyl-3-[[1-(3,4-dichlorophenyl)tetrazol-5-yl]methylsulfanyl]-4H-1,2,4-triazin-5-one	Yes	Yes	Yes	Yes	Yes	Yes
28	ZINC000009304125	6-benzyl-3-[[1-(4-chlorophenyl)tetrazol-5-yl]methylsulfanyl]-4H-1,2,4-triazin-5-one	Yes	Yes	Yes	Yes	Yes	Yes
29	ZINC000013627408	6-benzyl-3-[(1-phenyltetrazol-5-yl)methylsulfanyl]-4H-1,2,4-triazin-5-one	Yes	Yes	Yes	Yes	Yes	Yes
30	ZINC000009304125	6-benzyl-3-[[1-(4-chlorophenyl)tetrazol-5-yl]methylsulfanyl]-4H-1,2,4-triazin-5-one	Yes	Yes	Yes	Yes	Yes	Yes
31	ZINC000009302601	6-benzyl-3-[[1-[3-(trifluoromethyl)phenyl]tetrazol-5-yl]methylsulfanyl]-4H-1,2,4-triazin-5-one	Yes	Yes	Yes	Yes	Yes	Yes
32	ZINC000013627512	6-phenyl-3-[[1-[3-(trifluoromethyl)phenyl]tetrazol-5-yl]methylsulfanyl]-4H-1,2,4-triazin-5-one	Yes	Yes	Yes	Yes	Yes	Yes
33	ZINC000013625488	3-[[1-(4-chlorophenyl)tetrazol-5-yl]methylsulfanyl]-6-phenyl-4H-1,2,4-triazin-5-one	Yes	Yes	Yes	Yes	Yes	Yes
34	ZINC000009418994	6-phenyl-3-[(1-phenyltetrazol-5-yl)methylsulfanyl]-4H-1,2,4-triazin-5-one	Yes	Yes	Yes	Yes	Yes	Yes

Sixteen of the 34 molecules satisfied all the requirements of all the five drug-likeness filters.

## Data Availability

The data supporting the findings of this study are available upon request from the corresponding author.
